# Sphingosine-1 Phosphate Lyase Regulates Sensitivity of Pancreatic Beta-Cells to Lipotoxicity

**DOI:** 10.3390/ijms221910893

**Published:** 2021-10-08

**Authors:** Yadi Tang, Thomas Plötz, Markus H. Gräler, Ewa Gurgul-Convey

**Affiliations:** 1 Institute of Clinical Biochemistry, Hannover Medical School, 30625 Hannover, Germany; Yadi.Tang@stud.mh-hannover.de (Y.T.); Ploetz.Thomas@mh-hannover.de (T.P.); 2 Department of Anesthesiology and Intensive Care Medicine, University Hospital Jena, 07740 Jena, Germany; Markus.Graeler@med.uni-jena.de

**Keywords:** sphingosine-1 phosphate, sphingosine-1 phosphate lyase, insulin-secreting cells, diabetes, lipotoxicity, human beta-cells

## Abstract

Elevated levels of free fatty acids (FFAs) have been related to pancreatic beta-cell failure in type 2 diabetes (T2DM), though the underlying mechanisms are not yet fully understood. FFAs have been shown to dysregulate formation of bioactive sphingolipids, such as ceramides and sphingosine-1 phosphate (S1P) in beta-cells. The aim of this study was to analyze the role of sphingosine-1 phosphate lyase (SPL), a key enzyme of the sphingolipid pathway that catalyzes an irreversible degradation of S1P, in the sensitivity of beta-cells to lipotoxicity. To validate the role of SPL in lipotoxicity, we modulated SPL expression in rat INS1E cells and in human EndoC-βH1 beta-cells. SPL overexpression in INS1E cells (INS1E-SPL), which are characterized by a moderate basal expression level of SPL, resulted in an acceleration of palmitate-mediated cell viability loss, proliferation inhibition and induction of oxidative stress. SPL overexpression affected the mRNA expression of ER stress markers and mitochondrial chaperones. In contrast to control cells, in INS1E-SPL cells no protective effect of oleate was detected. Moreover, *Plin2* expression and lipid droplet formation were strongly reduced in OA-treated INS1E-SPL cells. Silencing of SPL in human EndoC-βH1 beta-cells, which are characterized by a significantly higher SPL expression as compared to rodent beta-cells, resulted in prevention of FFA-mediated caspase-3/7 activation. Our findings indicate that an adequate control of S1P degradation by SPL might be crucially involved in the susceptibility of pancreatic beta-cells to lipotoxicity.

## 1. Introduction

Type 2 diabetes (T2DM) affects the majority of patients with diabetes and is triggered by unhealthy diet and physical inactivity together with a genetic predisposition [1,2]. Elevated plasma free fatty acid (FFA) levels have been detected at the prediabetes state and in T2DM patients, and it is believed that chronic exposure of pancreatic insulin-secreting beta-cells to FFAs induce beta-cell dysfunction and death [3,4,5,6]. FFAs induce a multimodal stress response, including oxidative stress, endoplasmic reticulum (ER) stress and mitochondrial dysfunction, leading to dysfunction and death of beta-cells [3,6,7,8,9,10,11,12]. The two most abundant dietary FFAs in plasma are saturated palmitic acid (PA) and monounsaturated oleic acid (OA)—of which, OA exerts toxic effects in human pancreatic beta-cells [13,14], in contrast to rodent beta-cells in which it protects from PA-mediated cell death [15]. The underlying mechanisms of the broader sensitivity of human beta-cells to a variety of FFAs are not fully understood. Recently, stearoyl-CoA desaturase 1 (SCD1), an ER enzyme that is involved in monounsaturated fatty acid synthesis from PA and stearic acid (SA), was shown to be abundantly expressed in human beta-cells [9]. PA was shown to downregulate SCD1 expression and siRNA-mediated silencing was shown to induce beta-cell death [9]. These findings indicate that the intracellular capacity to decrease biological availability of PA for various biosynthetic or metabolic pathways may be crucially involved in the protection of human beta-cells against lipotoxicity.

Palmitate serves as a starting point for the biosynthesis of sphingolipids (SLs) [16]. Accumulating evidence points to an important role of SLs, particularly ceramides, in the regulation of beta-cell dysfunction and death during T2DM development [5,11,17,18,19,20,21,22,23]. SLs are ubiquitous, structurally diverse lipid species that are essential components of cell membranes and bioactive mediators regulating cell fate and metabolism. The complex SL pathway begins in the ER from the reaction of palmitoyl-CoA with L-serine catalyzed by serine-palmitoyl-transferase (SPT) [16]. SPT is characterized by a high specificity for the CoA-thioester of PA [24], and therefore, the flux of de novo SL biosynthesis strongly depends on the availability of PA. A series of enzymatic reactions leads to formation of a central SL ceramide, which is a precursor of sphingosine and complex SLs [16]. Sphingosine is a substrate for the generation of sphingosine-1 phosphate (S1P) [25]. This reaction is catalyzed by two types of sphingosine kinases (SK), of which, SK2—which raises S1P levels in the mitochondria, ER and nucleus—is the predominant isoform in pancreatic beta-cells [23,26]. S1P can be dephosphorylated by the actions of S1P-specific phosphatases (SPPs), or by the nonspecific lipid phosphate phosphatase family (LPPs) [27]. The last step in sphingolipid metabolism is the irreversible degradation of S1P to hexadecenal and phosphoethanolamine by the highly conserved ER enzyme S1P lyase (SPL) [28]. Rodent beta-cells are characterized by a moderate expression of SPL [26]. The SPL-reaction products can be transferred to the glycerophospholipid pathway and hexadecenal can be used for reloading the palmitoyl-CoA pool [29]. An insufficient degradation of hexadecenal leads to its accumulation and elicits cellular toxicity [30,31].

S1P is a potent modulator of cellular function and fate in various cell types, including pancreatic beta-cells [11,16,18,20,21,25,27,32,33,34]. The serum and tissue concentrations of S1P have been shown to be elevated in the metabolic state preceding T2DM [35]. A high fat and sugar diet as well as obesity have recently been linked to activation of SK/S1P/S1P receptor signaling and induction of inflammation in many gastrointestinal disorders, including cancer [36]. S1P can act extracellularly by activation of cell surface S1P receptors (S1PR1-5) or intracellularly as a second messenger and epigenetic modulator [25]. Activation of SK1 in parallel with increased S1P generation has been shown to regulate cell proliferation and stem cell differentiation [36,37], while SPL was shown to regulate stem cell cycle quiescence [38].

PA was shown to induce ceramide and dihydro-S1P, but not S1P accumulation under prolonged hyperglycemic conditions (24 h) in INS1 insulin-secreting cells [23]. Loss of SK1 was shown to predispose to diabetes development via promotion of beta-cell death in diet-induced obese mice [39]. Pharmacological suppression of SK1 in INS1 cells was associated with acceleration of PA toxicity, while overexpression of SK1 protected beta-cells against PA-mediated apoptosis [23]. The protective effect of SK1 overexpression was related to the intracellular action of S1P and correlated with decreased levels of proapoptotic ceramides [23]. In contrast, a negative role of SK2 was described in PA-treated rodent beta-cells and in a mouse model of T2DM [40,41]. This toxic effect was related to shuttling of SK2 from the nucleus to the cytoplasm and correlated with mitochondrial cell death [41].

Thus, an accumulating body of evidence indicates that the biosynthesis of S1P may be crucially involved in the sensitivity of beta-cells to PA. So far, the role of S1P degradation capacity in the toxic effects of FFAs in beta-cells has not been addressed. Therefore, in the present study we undertook the question of whether SPL may be engaged in the regulation of beta-cell sensitivity to FFAs. We demonstrate that SPL overexpression boosts lipotoxicity in rodent beta-cells and show that the significantly higher SPL expression in human beta-cells correlates with their broad-range sensitivity to FFAs.

## 2. Results

### 2.1. SPL Overexpression in Insulin-Secreting INS1E Cells Downregulates Intracellular S1P

**Figure 1:**
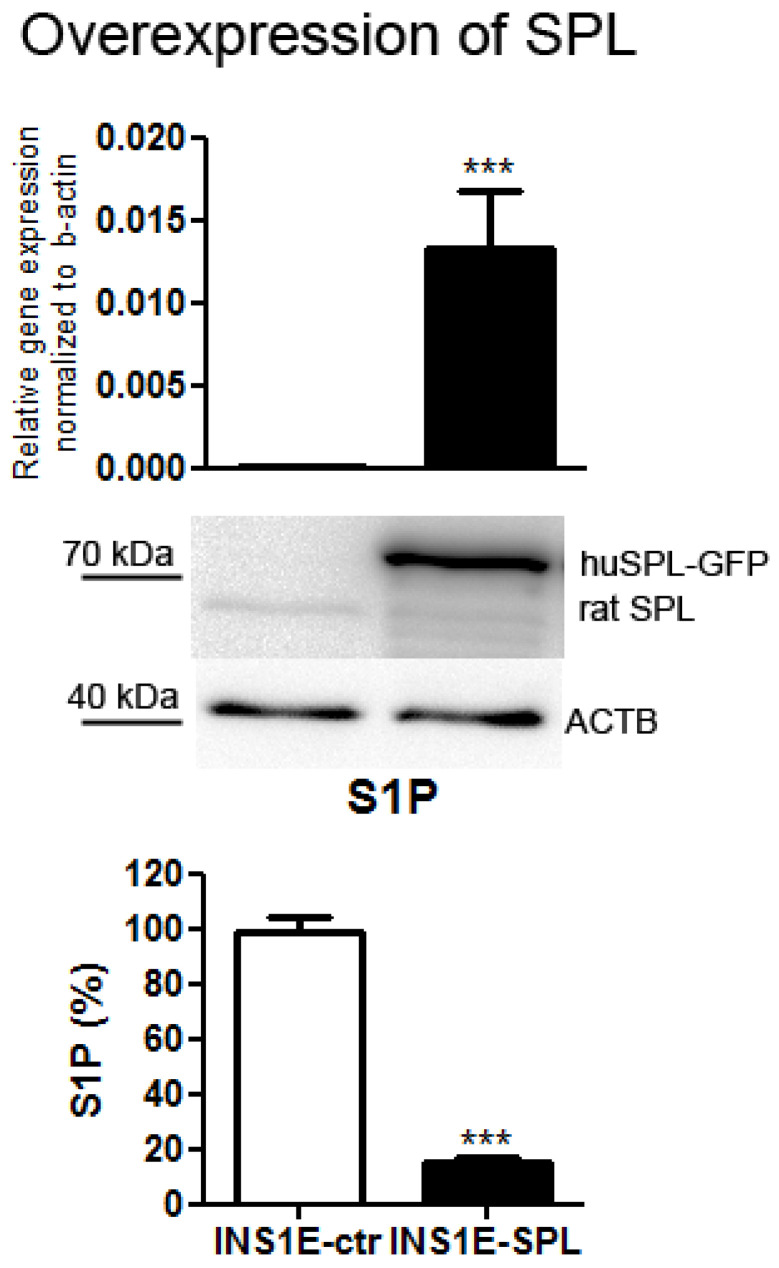
Overexpression of human SPL in rat INS1E insulin-secreting cells. INS1E cells were stably transfected with the pcDNA3-huSPL-GFP construct using lipofectamine. Positive clones were selected by resistance to G418. Confirmation of SPL expression level was performed on the mRNA (real-time PCR) and protein levels (Western blot using an antibody enabling detection of rat and human SPL) as well as by measurements of intracellular S1P by ESI-LC–MS/MS (S1P in INS1E-control cells was set as 100%). Shown are MEANS ± SEM from 4 independent samples. *t*-test, *** *p* < 0.001 vs. INS1E-control cells. The magnitude of SPL overexpression was regularly checked during the entire time of cell culture. Visible are weak rat SPL protein bands of 63 kDa and a strong band of human SPL-GFP. Beta-actin was used as a loading control (ACTB).

To address the importance of a high S1P degradation capacity and SPL for FFA effects in beta-cells we chose rat insulin-secreting INS1E cells, which are an established, robust model for studies of molecular mechanisms underlying the effects of various diabetogenic substances, including FFAs. We formerly showed that the expression of SPL in INS1E cells very closely resembles the expression of SPL in primary rat islets [26]. Comparing to other tissues, SPL expression is in a medium–low range, which makes these cells a good model for SPL overexpression [26]. By means of a lipofectamine-mediated stable transfection we generated INS1E cells overexpressing human SPL tagged with GFP, enabling easy tracking of overexpression stability during routine cell culture (Figure 1). The expression of SPL was significantly elevated, as revealed by measurements of mRNA and protein levels (Figure 1). SPL overexpression resulted in a strong decrease in S1P concentration by around 80% as compared to INS1E-ctr cells (Figure 1), demonstrating that the SPL-GFP was enzymatically active.

### 2.2. SPL Overexpression Sensitizes Insulin-Secreting INS1E Cells to FFA-Mediated Viability Loss

**Figure 2:**
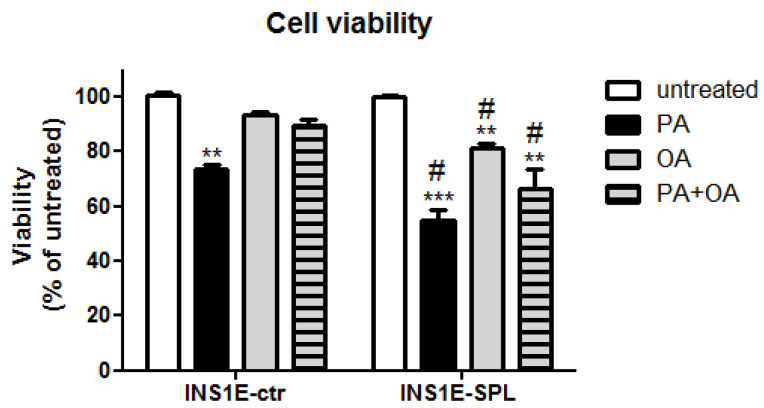
Effects of SPL overexpression and free fatty acids on cell viability in rat INS1E insulin-secreting cells. INS1E cells were incubated in the absence or presence of 500 µM PA, OA or a combination of PA and OA (500 µM of each) for 24 h. Thereafter, cell viability was measured by MTT assay. Shown are MEANS ± SEM from n = 6 independent experiments; each condition was measured in triplicate. ANOVA followed by Bonferroni. ** *p* < 0.01, *** *p* < 0.001 vs. untreated, # *p* < 0.05 vs. INS1E-ctr cells treated in the same way.

To study the influence of SPL overexpression on the susceptibility of INS1E cells to FFAs we incubated cells for 24 h with 500 µM PA, 500 µM OA or with a combination of PA + OA (each at the concentration of 500 µM). This concentration was chosen based on our earlier studies and concentration-dependency experiments [4,14,15]. Cell viability was estimated using a MTT assay, the results of which correlate with cellular metabolic activity and which is commonly used as an indicator of cell viability, proliferation and cytotoxicity [42]. A 24 h exposure to PA resulted in a 30% decrease in cell viability in INS1E-ctr cells (Figure 2). OA did not induce a significant drop in cell viability and prevented PA-induced cell viability loss in INS1E-ctr cells (Figure 2). Interestingly, overexpression of SPL strongly potentiated PA-mediated cell viability loss (Figure 2). Moreover, in INS1E-SPL cells, incubation with OA resulted in a significant cell viability decrease and the protective effect of OA on PA-toxicity was lost (Figure 2).

### 2.3. SPL Overexpression Potentiates FFA-Mediated Proliferation Inhibition

**Figure 3:**
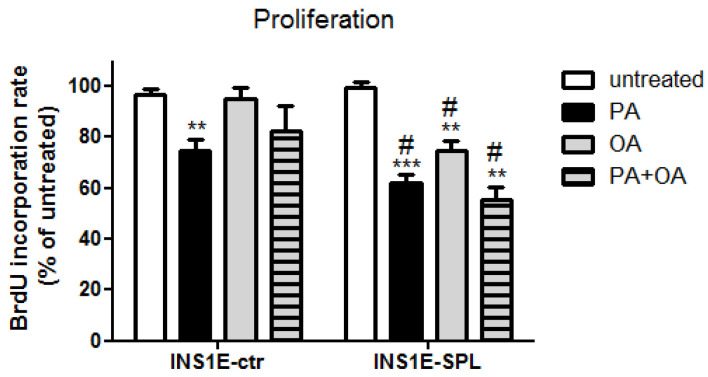
Effects of SPL overexpression and free fatty acids on proliferation rates in rat INS1E insulin-secreting cells. INS1E cells were incubated in the absence or presence of 500 µM PA, OA or a combination of PA and OA (500 µM of each) or 24 h. Thereafter, proliferation rate was estimated by BrdU incorporation. Shown are MEANS ± SEM from n = 6 independent experiments; each condition was measured in triplicate. ANOVA followed by Bonferroni, ** *p* < 0.01, *** *p* < 0.001 vs. untreated, # *p* < 0.05 vs. control cells treated in the same way.

Next, we analyzed the effects of SPL overexpression on the proliferation rate of INS1E cells exposed to FFAs. In INS1E-ctr cells we observed a significantly lower incorporation rate of BrdU, indicating a slower proliferative capacity (Figure 3). Again, OA alone or in combination with PA was not associated with any deleterious effect regarding cell proliferation (Figure 3). This was in contrast to SPL-overexpressing INS1E cells, which were characterized by a significantly stronger inhibition of proliferation induced by PA and a decrease of proliferation in response to OA or PA + OA (Figure 3).

### 2.4. SPL Overexpression Potentiates FFA-Mediated Oxidative Stress

**Figure 4:**
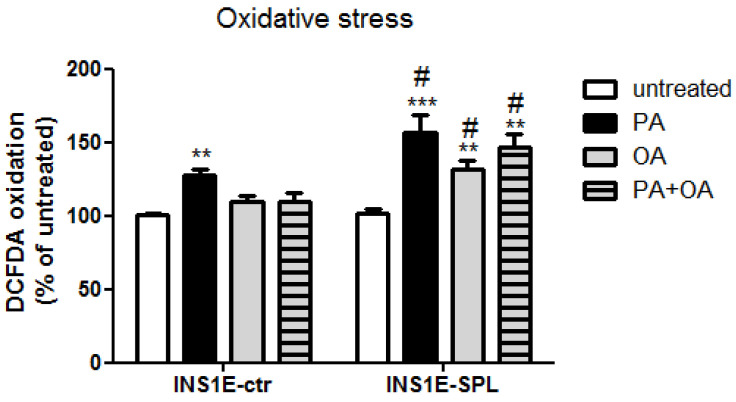
Effects of SPL overexpression and free fatty acids on oxidative stress in rat INS1E insulin-secreting cells. INS1E cells were incubated in the absence or presence of 500 µM PA, OA or a combination of PA and OA (500 µM of each) for 24 h. Thereafter, oxidative stress was estimated by DCFDA oxidation rate. Shown are MEANS ± SEM from n = 6 independent experiments; each condition was measured in triplicate. ANOVA followed by Bonferroni, ** *p* < 0.01, *** *p* < 0.001 vs. untreated, # *p* < 0.05 vs. INS1E-ctr cells treated in the same way.

FFA-mediated toxicity in pancreatic beta-cells has been shown to be associated with an increased generation of reactive oxygen species (ROS), particularly in peroxisomes and in the cytoplasmic compartment [4,15,43]. We estimated oxidative stress using a robust DCFDA-based assay, which enables detection of changes in overall ROS generation. In line with earlier findings, we observed a significant induction of oxidative stress in INS1E-ctr cells exposed to PA, but not to OA (Figure 4). Interestingly, SPL overexpression was associated with a significantly stronger induction of ROS generation in PA-treated cells as compared to INS1E-ctr cells (Figure 4). Similarly, incubation with OA resulted in an increased ROS generation in INS1E-SPL cells (Figure 4).

### 2.5. SPL Overexpression Regulates the ER Stress Response under PA Challenge

**Figure 5:**
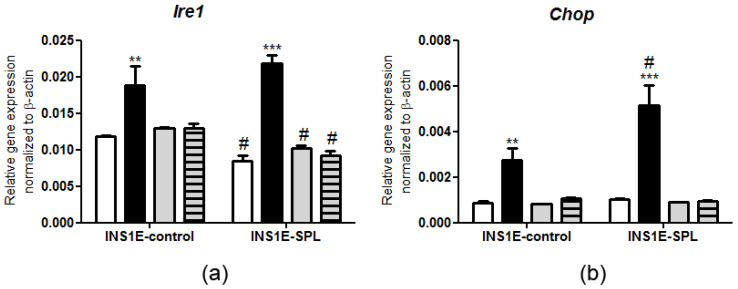
Effects of SPL overexpression and free fatty acids on ER stress markers expression. INS1E cells were incubated in the absence or presence of 500 µM PA, OA or a combination of PA and OA (500 µM of each) for 24 h. Thereafter, RNA was isolated, cDNA was synthetized and real-time PCR was performed to detect (**a**) *Ire1* and (**b**) *Chop* expression. Shown are MEANS ± SEM from n = 4–6 independent experiments; each condition was measured in triplicate. Open bars: untreated, black bars: PA, grey bars: OA, grey striped bars: PA+OA. ANOVA followed by Bonferroni ** *p* < 0.01, *** *p* < 0.001 vs. untreated, # *p* < 0.05 vs. INS1E-ctr cells treated in the same way.

Elevated concentrations of FFAs have been shown to induce the ER stress response in beta-cells [7,23,44,45,46], which has serious consequences for beta-cell function and fate. We analyzed the gene expression of two ER stress markers, namely the key UPR signaling activator Ire1 and the crucial transcription factor mediating cell death Chop. Previously, it has been demonstrated that in rat INS1E cells PA is a particularly strong inducer of Ire1 and Chop expression [44], and these findings were confirmed in our experiments (Figure 5). SPL overexpression resulted in a lower expression of *Ire1* (Figure 5a). The magnitude of PA-mediated upregulation of *Ire1* expression was stronger in INS1E-SPL cells as compared to INS1E-ctr cells (Figure 5a). The basal *Chop* expression was not affected by SPL overexpression, but the PA-mediated induction of *Chop* was potentiated (Figure 5b). Thus, SPL overexpression may be involved in the regulation of the gene expression of ER stress markers in beta-cells.

### 2.6. SPL Overexpression Regulates the Expression of Mitochondrial Chaperones in Response to FFAs

**Figure 6:**
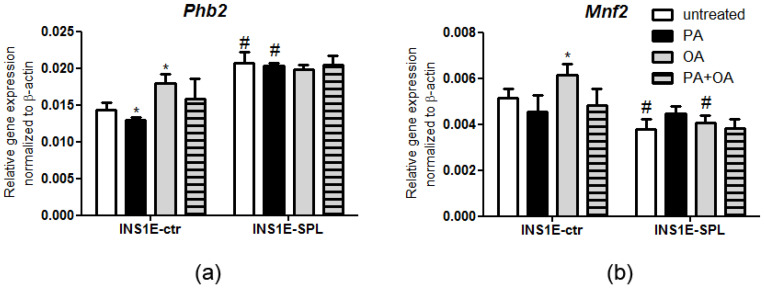
Effects of SPL overexpression and free fatty acids on mitochondrial stress markers expression. INS1E cells were incubated in the absence or presence of 500 µM PA, OA or a combination of PA and OA (500 µM of each) for 24 h. Thereafter, RNA was isolated, cDNA was synthetized and real-time PCR was performed to detect (**a**) *Phb2* and (**b**) *Mnf2* expression. Shown are MEANS ± SEM from n = 4–6 independent experiments; each condition was measured in triplicate. ANOVA followed by Bonferroni, * *p* < 0.05 vs. untreated, # *p* < 0.05 vs. INS1E-ctr cells treated in the same way.

Mitochondria are the site of beta-oxidation of FFAs, ROS generation and ATP biosynthesis. Mitochondrial stress has been well documented in beta-cells during T2DM development [6,10]. In our present study, we analyzed the effects of SPL overexpression upon FFA exposure on the expression of two mitochondrial chaperones that have been shown to participate in oxidative stress, ATP synthesis regulation and mitochondrial morphology. First, we analyzed the expression of prohibitin 2 (Phb2), an inner mitochondrial membrane protein that is involved in the regulation of mitophagy and ATP biosynthesis [47,48]. Confirming our earlier observations [26] we observed a higher basal expression of *Phb2* in INS1E-SPL cells as compared to INS1E-ctr cells (Figure 6a). In control cells, incubation with PA led to a small reduction in *Phb2* expression, while OA significantly stimulated mRNA expression of *Phb2* (Figure 6a)—in line with a protective effect of OA. FFAs did not modulate the gene expression of *Phb2* in INS1E-SPL cells (Figure 6a).

Next, we estimated the effects of FFAs on the gene expression of mitofusin 2 (Mnf2), a mitochondrial protein essential for mitochondrial fusion and maintenance of mitochondrial morphology [49]. Incubation with OA resulted in a significant increase of *Mnf2* expression in INS1E-ctr cells, but not in INS1E-SPL cells (Figure 6b). INS1E-SPL cells were characterized by a lower *Mnf2* gene expression level than INS1E-ctr cells (Figure 6b). Thus, SPL overexpression may regulate the expression of mitochondrial chaperones in response to FFAs in beta-cells.

### 2.7. SPL Overexpression Inhibits Lipid Droplet Formation

**Figure 7:**
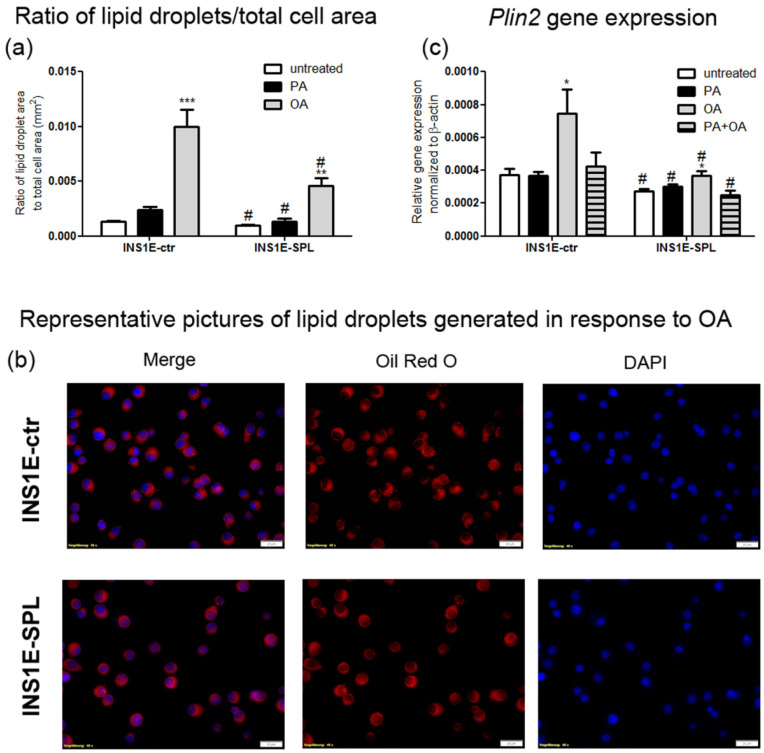
Effects of SPL overexpression on free fatty acid-mediated lipid droplet formation and *Plin2* gene expression. INS1E cells were stably transfected with the pcDNA3-huSPL-GFP vector, followed by clone selection by G418. Cells were incubated in the absence or presence of 500 µM PA, OA or a combination of PA and OA (500 µM of each) for 24 h. Thereafter, (**a**) cells were stained with Oil Red O and DAPI (nuclear staining), fixed and analyzed using a CellR/Olympus IX81 inverted microscope system (objective 40×, Olympus, Hamburg, Germany). To quantify lipid droplet formation in single cells, images were analyzed with xCellence Rt software. The fluorescence of lipid droplets is shown in relation to the total cell area and measured at 560/630 nm, and representative pictures are shown in (**b**) (red: Oil Red O, blue: DAPI, bar: 20 µm). Shown in (**c**) is the mRNA expression of *Plin2*. RNA was isolated, cDNA was synthetized and real-time PCR was performed. Shown are MEANS ± SEM from n = 4–6 independent experiments; each condition was measured in triplicate. ANOVA followed by Bonferroni, * *p* < 0.05, ** *p* < 0.01, *** *p* < 0.001 vs. untreated, # *p* < 0.05 vs. INS1E-ctr cells treated in the same way.

Lipid droplets (LDs) are storage units for various lipids, including ceramides [50,51]. The formation of LDs has been shown to be induced by unsaturated FFAs in beta-cells [52] and some studies indicate its protective role against saturated FFA-mediated toxicity [53]. To address the question of whether an observed potentiation of FFA toxicity by SPL overexpression may be related to disturbances in lipid droplet formation, we analyzed LD generation by Oil Red O staining and by gene expression measurements of perilipin 2 (Plin2), a protein highly expressed in LDs and involved in their biosynthesis [51,54,55]. As expected, we observed a significant, strong induction of LD formation in response to OA in INS1E-ctr cells, as shown by an increased ratio of LD area/total cell area and by the presence of multiple LD puncta in cells (Figure 7a,b). These observations went in line with a stimulation of *Plin2* expression in OA-treated INS1E-ctr cells (Figure 7c). Interestingly, we detected a significantly lower number of LDs in INS1E-SPL cells as compared to INS1E-control cells (Figure 7a,b). Consistent with this finding, the expression of *Plin2* was significantly lower in INS1E-SPL cells in comparison to INS1E-ctr cells and was only slightly induced by OA (Figure 7c). Thus, changes in intracellular S1P concentration may be involved in the regulation of lipid droplet formation in beta-cells.

### 2.8. SPL Knockdown Protects Human EndoC-βH1 Cells from FFA-Mediated Toxicity

**Figure 8:**
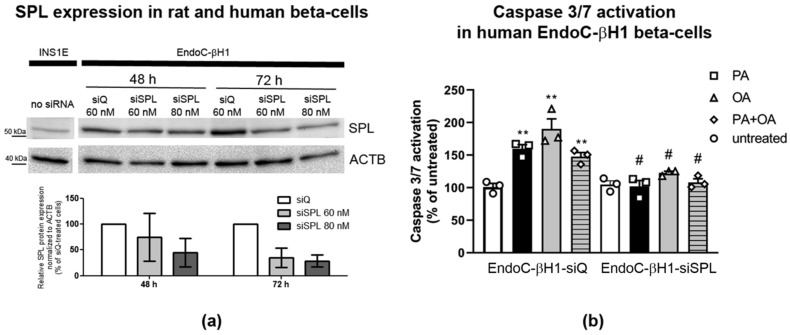
Knockdown of SPL in human EndoC-βH1 beta-cells and its consequences for FFA-induced caspase-3/7 activation. Shown are (**a**) Western blot analysis of SPL expression in rat INS1E cells and human EndoC-βH1 beta-cells. Human EndoC-βH1 beta-cells were transiently transfected with either control siRNA (no specific target, siQ) or with Silencer^®^Select RNAi targeted against human SPL (ThermoFisher Scientific, Braunschweig, Germany). Two concentrations of siRNA and two different times post-transfection (48 and 72 h) were tested twice prior to planned apoptosis assays to validate the best conditions for efficient SPL silencing. All experimental samples and controls were run on the same blot image. A representative blot of 2 independent experiments and the densitometry analysis of both experiments (as % of siQ-treated cells) are shown; (**b**) For subsequent experiments on the role of SPL knockdown on FFA-mediated apoptosis induction, EndoC-βH1 beta-cells were transfected with 80 nM siRNA and 72 h post-transfection incubated in the absence or presence of 500 µM PA, OA or a combination of PA and OA for 24 h. Thereafter, caspase-3/7 activation was measured by caspase3/7-Glo assay (Promega, Walldorf, Germany). Shown are MEANS ± SEM and single values (as dot symbols, each dot represents one individual experiments) from n = 3 independent experiments; each condition in each experiment was measured at least in triplicate. Open bars: untreated, black bars: PA, grey bars: OA, grey striped bars: PA + OA. ANOVA followed by Bonferroni, ** *p* < 0.01 vs. untreated, # *p* < 0.05 vs. EndoC-βH1 siQ beta-cells treated in the same way.

Our results strongly suggest that SPL might be involved in the sensitivity of beta-cells to FFAs. It is, meanwhile, well established that human beta-cells exhibit divergent responses to FFAs and their toxic effects [3,7,9,14,15,56] compared to rodent beta-cells. Importantly, monounsaturated FFAs (such as OA) that protect rodent insulin-secreting cells against saturated FFA-mediated toxicity (such as PA) induce apoptosis in human beta-cells [13]. To address the question of whether SPL may be involved in toxic effects of FFAs in human beta-cells, we used a well characterized human EndoC-βH1 beta-cell line, which is very close in phenotype to native adult human beta-cells [57,58]. Using an antibody that specifically detects rodent and human SPL protein, we analyzed the expression of SPL in rat insulin-secreting INS1E cells and in human EndoC-βH1 beta-cells (Figure 8a). We observed that human beta-cells express SPL on a significantly higher protein level than rat insulin-secreting INS1E cells (Figure 8a). In the next step, we determined whether suppression of SPL in human EndoC-βH1 beta-cells may affect the susceptibility of these cells to FFAs. We transiently transfected human EndoC-βH1 beta-cells either with a control, non-targeting siRNA or with a validated Silencer^®^Select RNAi against human SPL, and verified the optimal conditions for the most effective suppression of SPL (Figure 8a). For the analysis of FFA-induced apoptosis we used cells 72 h after transfection with 80 nM Silencer^®^Select RNAi, which resulted in >80% reduction of SPL protein expression and was characterized by the lowest level of efficiency variability (Figure 8a).

Exposure of control EndoC-βH1-siQ beta-cells to FFA-induced caspase 3/7 activation (Figure 8b). PA (500 µM) increased caspase-3/7 activation by approximately 150%, while OA used at the same concentration elevated it by nearly 200% (Figure 8b). No additive effect of FFAs when used in combination was observed (Figure 8b). Strikingly, silencing of SPL prevented PA-mediated caspase-3/7 activation in EndoC-βH1-siSPL beta-cells (Figure 8b). Moreover, the OA-induced caspase-3/7 activation was significantly blunted by SPL knockdown (Figure 8b). Consequently, caspase 3/7 activation in response to PA + OA was also significantly reduced in EndoC-βH1-siSPL beta-cells (Figure 8b). Thus, SPL silencing prevented FFA-mediated caspase-3/7 activation in human beta-cells.

## 3. Discussion

The rising burden of T2DM is a serious concern for healthcare systems worldwide [1,2]. T2DM is preceded by the prediabetes state, coupled with glucose intolerance, systemic inflammation and hyperlipidemia [1,2]. Elevated serum concentrations of FFAs have been associated with pancreatic beta-cell dysfunction and impaired glucose-stimulated insulin secretion [3,12,59]. Chronic exposure to high concentrations of FFAs was shown to stimulate toxic effects in beta-cells—a phenomenon called lipotoxicity [1,2,3,6,12]. So far, the mechanisms of lipotoxicity in pancreatic beta-cells have been extensively studiedmainly in rodent insulin-secreting beta-cell lines and islets due to a scarce supply of human islets. Numerous groups have demonstrated that toxic effects of FFAs in beta-cells involve an induction of oxidative stress, activation of ER stress and mitochondrial dysfunction [3,6,7,8,9,10,11,12]. The generation of the human EndoC-βH1 beta-cell line [57] has enabled studies on the specific effects of various FFAs in human beta-cells [13,14,56,60,61]. These studies confirmed and extended the observations gained from human islets, which demonstrated that human beta-cells are vulnerable to a variety of FFAs, including the most abundant monounsaturated FA: oleic acid [13,14,56]—which in rodent beta-cells exerts protective effects against toxic saturated FFAs [15,62]. The underlying mechanisms of lipotoxicity in human beta-cells remain unclear. Recently, an important role of SCD1 as a gatekeeper of human beta-cell phenotype and lipotoxicity has been described [9]. SCD1 is an ER-localized enzyme, which uses saturated palmitic acid (PA) to produce the monounsaturated palmitoleic acid [63], and as such, SCD1 could downregulate the intracellular pool of PA available for other biosynthetic or harmful metabolic pathways. However, a diminished islet expression of SCD1 has been associated with T2DM development and it is believed that this reduced SCD1 expression may render beta-cells more susceptible to PA-induced ER stress and apoptosis [9,64].

Palmitate is a main substrate for biosynthesis of sphingolipids. Remarkable changes in SL serum and tissue profiles have been described in T2DM patients [35,65], and SLs, particularly ceramides, are believed to be crucially involved in the development of insulin resistance and T2DM as well as beta-cell dysfunction [5,6,7,11,18,21,22,23,34,35,39,40,41,66].

Several groups studied the role of intracellular S1P in beta-cells by manipulation of the expression level of two S1P-generating enzymes: SK1 and SK2. Rodent pancreatic beta-cells are characterized by a higher expression level of SK2 than of SK1 [23,26]. The effects of upregulation of intracellular S1P generation capacity seem to depend on the site of S1P production. The beta-cell protective effect was observed by overexpression of SK1, which generates S1P at the plasma membrane and by ER-targeted SK1 overexpression [23]. The antiapoptotic effect of SK1 overexpression involves the inhibition of PA-induced ceramide synthesis [23]. A pharmacological inhibition of SK1 accelerated PA-induced cell death [23]. Additionally, loss of SK1 was shown to promote the onset of diabetes by accelerating beta-cell death in diet-induced obese mice [39]. In contrast, overexpression of SK2 has been associated with acceleration of PA toxicity [41], while silencing SK2 expression downregulated PA-stimulated cell death in INS1E cells and improved glucose homeostasis in a T2DM mouse model [41]. Thus, data gained from studying the role of SKs indicate that the intracellular concentration and the site of generation of S1P determine its effects on lipotoxic beta-cell death.

So far, the role of the S1P-degrading enzyme SPL has not yet been studied in context of lipotoxicity in beta-cells. SPL is the only exit point in the SL pathway and as such, controls not only the availability of S1P for S1P-dependent biological processes, but also regulates the flow of SL intermediates into phospholipid pathway [67]—thereby serving as a gatekeeper of lipid metabolic flow [67,68,69,70]. Products of the SPL reaction are involved in cell fate by distinct mechanisms; accumulation of hexadecenal has been shown to induce DNA breakage [30,71] and mitochondrial fragmentation [72], while phosphoethanolamine production has been shown to regulate accumulation of aggregate-prone proteins such as amyloid beta precursor polypeptide in neurons [73,74].

In our present study, we undertook characterization of the importance of SPL for beta-cell fate under lipotoxic conditions. Our former study revealed that the expression levels of SPL in rodent beta-cells is relatively low as compared to the expression of sphingosine kinases—particularly that of SK2 [26]. Therefore, rodent beta-cells serve as an excellent model for studying the effects of SPL overexpression on sensitivity to FFAs. Because SPL overexpression resulted in an approximately 80% decrease in S1P content, most of the observed effects are likely linked to its enzymatic activity and a lower biological availability of S1P. However, it cannot be excluded that increased SPL protein expression per se could also contribute.

The current study revealed an enhanced cell viability loss and proliferation inhibition in INS1E-SPL cells exposed to PA, which went along with a stronger induction of oxidative stress. Moreover, we observed that SPL overexpression also elicited toxic effects of OA, which is nontoxic in control INS1E cells [15]. The protection of OA against PA-induced cell death and ROS formation was lost in INS1E-SPL cells. The unexpected sensitization to OA observed in SPL-overexpressing cells could be related to the observed dramatic loss of LD formation in INS1E-SPL cells. LDs are storage units, mainly for neutral lipids especially triglycerides but also for proapoptotic ceramides [50,51,75]. Increased LD formation in liver parenchyma was described in SPL knockout mice [67]. The formation of LD has been shown to be induced by unsaturated FFAs in beta-cells and several studies have indicated their protective role against saturated FFA-mediated toxicity [53], although no consensus exists on their cytoprotective role in various models of beta-cells. In the present study, there was a correlation between enhanced FFA toxicity and the dramatically reduced LD area in INS1E-SPL cells, which went along with decreased expression of *Plin2*. Though the exact mechanism is unclear, former studies in other cell types such as hepatocytes indicate that SLs can influence LD formation [50,76]. Plin2-coated lipid droplets were shown before to inhibit FFA availability for mitochondria metabolism [77]. It could therefore be possible that the reduced *Plin2* expression and concomitant reduced LD formation under exposure to high concentrations of FFAs may lead to a higher availability of FFAs for mitochondrial metabolism, which could be associated with changes in the expression of mitochondrial chaperones and enhanced ROS formation in SPL-overexpressing cells. This reduced LD formation could also result in pathological intracellular, ectopic fat accumulation, as observed in other cell types [50,51,75]. Such an accumulation of FFAs—especially of longer-chain FFAs—could also be involved in the observed increase in ROS generation in INS1E-SPL, since long-chain FFA metabolism in peroxisomes was linked with FFA-induced oxidative stress in beta-cells [4].

In line with increased toxicity, we observed a potentiated expression pattern of *Ire1* and *Chop* in response to PA in INS1E-SPL cells as compared to control cells. At variance with the induction of viability loss, OA failed to induce *Ire1* or *Chop* expression in INS1E-SPL cells, in a similar way to control cells—which indicates that sensitization to OA in INS1E-SPL cells was rather not related to induction of ER stress.

Additionally to activation of ER stress, we observed alterations in the expression of mitochondrial chaperons in FFA-treated INS1E-SPL cells. Pancreatic beta-cell mitochondria are characterized by a strong expression of manganese superoxide dismutase (MnSOD), but inexplicably are very poorly equipped with an enzymatic defense system against hydrogen peroxide—which makes this organelle particularly vulnerable to stress [78]. Earlier studies from various cell types have demonstrated that various SLs can regulate mitochondrial function [72,79,80]. Our current observations in INS1E-SPL cells point to the role of intracellular S1P concentration for proper beta-cell mitochondrial integrity under lipotoxic conditions. We observed a significantly lower expression of mitofusin 2 (*Mnf2*) in INS1E-SPL cells. A similar observation has been made in mouse MIN6 beta-cells treated with a SK inhibitor, which resulted in a reduced Mnf1 expression [81]. Mnf2 is the predominant isoform of mitofusin in rodent beta-cells and has been shown to control mitochondrial morphology, fusion and mitophagy [49]. However, the exact mechanism by which intracellular S1P and SPL expression may be involved in the regulation of mitochondrial function in beta-cells may be more complex, since SPL overexpression significantly upregulated the expression of the mitochondrial chaperone *Phb2* in our study, while SK2 suppression has been associated with decreased prohibitin expression in MIN6 beta-cells [81]. PHB2 is a highly conserved mitochondrial protein, which regulates mitochondrial assembly and ATP biosynthesis, and has been shown to be an important modulator of glucose-induced insulin secretion [47,48]. The intramitochondrial interaction of SK2-derived S1P with PHB2 has been shown to be important for cytochrome-c oxidase assembly and mitochondrial respiration [82]. Thus, SPL may be an important element in the regulation of mitochondrial network and function in beta-cells under lipotoxic stress. One of the possible involved mechanisms could rely on an increased hexadecenal generation in SPL-overexpressing cells exposed to PA, as hexadecenal accumulation has been shown to diminish oxygen consumption rates and increase ROS production and mitochondrial fragmentation in yeast [72]. Another mechanism involved in SPL overexpression-mediated effects could be rearrangements of mitochondrial CerS6-derived C-16:0 sphingolipids that have been recently shown to bind the mitochondrial fission factor and thereby induce mitochondrial fragmentation and stress [80].

Finally, we observed that in contrast to rodent beta-cells SPL is abundantly expressed in human EndoC-βH1 beta-cells. Interestingly, this observation correlated with distinct susceptibility profiles to FFA-mediated toxicity of rodent vs. human beta-cells, respectively [9,13,14,56]. To determine whether the high expression of SPL is indeed involved in the high sensitivity of human beta-cells to FFAs, we suppressed SPL expression in human EndoC-βH1 beta-cells and exposed these cells to FFAs. Strikingly, we observed a strong, almost complete prevention of apoptosis induction in SPL-suppressing EndoC-βH1 beta-cells exposed to PA, OA or a combination of both FFAs. These findings strongly suggest that SPL may be crucially involved in the vulnerability of human beta-cells to FFAs. The SPL expression in beta-cells could be also influenced by various environmental factors, such as sex hormones. Indeed, a significant role of estrogens in sphingolipid signaling in beta-cells as well as in the prevention of accumulation of misfolded proinsulin, ER stress, mitochondrial damage and in regulation of beta-cell metabolism has been demonstrated [34,83,84]. Low estrogen levels in (pre)menopausal women could lead to increased SPL expression and potentiation of FFA-mediated beta-cell failure, as a lower SPL expression has been observed in (phyto)estrogen-treated breast cancer cells [85]. Thus, enhanced SPL expression in beta-cells could participate in the well-known high susceptibility of (pre)menopausal women to T2DM development [86].

Though further studies are needed to establish the role of SPL in long-term effects of FFAs in human beta-cells, particularly under hyperglycemic conditions, inhibition of SPL might represent a promising pharmacological tool for future T2DM therapeutic approaches to protect beta-cells under lipotoxic stress.

## 4. Materials and Methods

### 4.1. Chemicals

Biotherm^TM^ Taq polymerase was from GeneCraft (Münster, Germany). Hybond N nylon membranes and the ECL detection system were from Amersham Biosciences (Freiburg, Germany). All other reagents were from Sigma Chemicals (Munich, Germany).

### 4.2. Cell Culture and FFA Incubations

Rat insulin-secreting INS1E cells (a kind gift of Prof. C.Wollheim, Geneva, Switzerland) and human EndoC-βH1 beta-cells (ENDOCELLS SARL, Paris, France) were cultured in a humidified atmosphere at 37 °C and 5% CO_2_. The cell lines used were routinely checked for mycoplasma and were free from mycoplasma contamination. INS1E cells were cultured in RPMI 1640 medium supplemented with 10 mM glucose, 10% fetal calf serum (FCS), penicillin and streptomycin, 10 mM Hepes (Serva, Heidelberg, Germany), 2 mM glutamine, 1 mM sodium-pyruvate (Sigma-Aldrich, Munich, Germany), and 50 μM of 2-mercaptoethanol [26]. EndoC-βH1 beta-cells were cultured onto coated dishes or plates (fibronectin and ECM) in a DMEM cell culture medium (5.5 mM glucose) without serum, but supplemented with 2% BSA, penicillin and streptomycin, 10 µM nicotinamide, 2.5 µg/mL transferrin, 6.7 ng/mL sodium selenite and 50 μM of 2-mercaptoethanol as described earlier [57,58]. For tests, cells were washed with PBS, followed by incubation with FFA (palmitate, oleate or in combination) at a concentration of 500 µM for 24 h. FFA incubations were performed in the presence of 1% FCS in the case of INS1E cells and under binding of FFA to BSA as described earlier [13,14,15,52]. The stock solutions of PA and of OA (50 mM) were freshly prepared using 90% ethanol as a solvent at 62 °C. The ratio between NEFA and BSA was 0.5 mM NEFA to 1% BSA.

### 4.3. Overexpression of Human SPL in Rat Insulin-Secreting INS1E Cells

The human SPL cDNA (pcDNA3.1-hSPLvector) [71] was stably overexpressed in insulin-secreting INS1E cells using the Lipofectamine™ transfection method. Positive clones were selected based on G418 and SPL expression levels confirmed by real-time PCR measurements, Western blotting and S1P measurements.

### 4.4. Suppression of Human SPL in EndoC-βH Beta-Cells

EndoC-βH1 beta-cells were transiently transfected with validated Silencer^®^Select RNAi against human SPL (assay S16965, ThermoFisher Scientific, Braunschweig, Germany) using Lipofectamine RNAiMax in OptiMEM medium. Silencer^®^Select RNAi were functionally tested and guaranteed a superior > 70% knockdown of the the target gene without off-target effects. 24 h after transfection cell culture the medium was changed. We tested 60 and 80 nM concentration of siRNA at two different times after transfection, namely 48 h and 72 h. Higher siRNA concentration and longer time post-transfection were associated with a stronger silencing effect, as shown in Figure 8a. This condition was used in the experiments analyzing the effects of FFAs on apoptosis induction.

### 4.5. Detection of S1P by ESI-LC–MS/MS

Lipids were extracted from cell pellets as described earlier [87]. Cell samples were used directly after adding 10 μL of the internal standard 10 μM C17-S1P (Avanti, Alabaster, AL, USA). Volumes of 300 μL 18.5% HCl, 1 mL methanol, and 2 mL chloroform were added to the glass centrifuge tubes and vortexed for 10 min. After centrifugation at 1900× *g* for 3 min, the lower chloroform phases were transferred into new centrifuge tubes and 2 mL chloroform added to the remaining aqueous phase. The vortexing and centrifugation steps were repeated, and the chloroform phases were combined. After evaporation of the solvent under vacuum for 45 min at 60 °C in a vacuum rotator, the dried samples were re-dissolved in 100 μL of a mixture containing 80% methanol and 20% chloroform and transferred into autosampler tubes and analysed with an LC–MS/MS QTrap triple quadrupole mass spectrometer (AB Sciex, Framingham, USA) coupled to a Hewlett Packard/Agilent Series1100 HPLC (Santa Clara, USA). Molecules were ionized by electrospray ionization (ESI) in the positive mode. The mass transitions for analysis in multiple reaction monitoring (MRM) mode were: S1P m/z 380/264 and C17-S1P m/z 366/250. Data analysis was done using Analyst 1.6.2 (AB Sciex, Framingham, MA, USA).

### 4.6. Viability Assay

Cells were seeded onto 96-well plates at a concentration of 30,000 cells/well and cultured for 48 h. Thereafter, cells were treated with FFAs for 24 h and cell viability was determined by a microplate-based MTT assay (3-(4,5-dimethylthiazol-2-yl)-2,5-diphenyl tetrazolium bromide, Serva, Heidelberg, Germany) as previously described [59]. The MTT assay is based on the conversion of MTT into formazan crystals by living cells, and detects total mitochondrial activity, which correlates with the number of cells for most cell populations.

### 4.7. Proliferation Assay

The proliferation rate of INS1E cells was quantified by a colorimetric method using the Cell Proliferation BrdU–ELISA (Roche, Mannheim, Germany). Cells were seeded at a concentration of 40,000 cells/well in 96-well microtiter plates and allowed to attach for 24 h. Thereafter, cells were incubated with the chemical compounds for 24 h. The proliferation assay was performed as described [26]. Absorbance was measured at 450 nm (reference wavelength 650 nm). Data were expressed as a percentage of untreated cells.

### 4.8. Detection of Caspase-3/7 Activation

After knockdown of SPL in EndoC-βH1 beta-cells, sensitivity to FFA-induced apoptosis was measured 24 h after FFA addition. The activation of the effector caspase-3/7 was analyzed by a 96-well-based chemiluminescence Caspase-3/7-Glo assay (Promega, Walldorf, Germany) according to the manufacturer’s protocol.

### 4.9. RNA Isolation, cDNA Synthesis and Real-Time RT-PCR

Total RNA from insulin-secreting cellswas obtained using an RNeasy Kit (Qiagen, Hilden, Germany). The quality of the total RNA was verified by agarose gel electrophoresis. RNA was quantified spectrophotometrically at 260/280 nm. Thereafter, 2 µg of RNA were reverse transcribed into cDNA using a random hexamer primer (Life Technologies, Carlsbad, CA, USA) and RevertAid H Minus M-MuLV reverse transcriptase (Thermo Fisher Scientific, Braunschweig, Germany). QuantiTect SYBR Green^TM^ technology (Qiagen), which uses a fluorescent dye that binds only double-stranded DNA, was employed. The reactions were performed on a ViiA7 real-time PCR system (Life Technologies) with the following protocol: 50 °C for 2 min, 95 °C for 10 min, and 40 cycles comprising a melting step at 95 °C for 15 s, an annealing step at 62 °C for 60 s and an extension step at 72 °C for 30 s. The quality of reactions was controlled by analysis of melting curves. Each sample was amplified in triplicate. Data were analyzed using the 2^−∆∆Ct^ method and normalized to the reference gene beta-actin. The primer sequences are given in Table 1. Primers were purchased from Microsynth (Balgach, Switzerland) and the efficiency was >90% for each primer set.

### 4.10. Western Blot Analysis

Cells were homogenized in ice-cold PBS containing protease inhibitors (Roche) using short bursts (Braun-Sonic 125 Homogenizer, Quigley-Rochester, Rochester, NY, USA). Protein content was determined by the BCA assay (Pierce). For Western blotting, 40 µg of total protein was resolved by SDS polyacrylamide gel electrophoresis and then electroblotted onto nitrocellulose membranes (GE Healthcare, Buckinghamshire, UK). Nonspecific binding sites of the membranes were blocked with 5% nonfat dry milk for 1 h at room temperature. The membranes were incubated with specific primary antibodies overnight at 4 °C. Immunodetection was performed using specific primary antibodies (goat anti-SP-lyase T-20 #sc-51431 diluted 1:500, Lot. No A1808 Santa Cruz, goat anti-actin C4 #sc47778, Santa Cruz, diluted at 1:1000) as described [26]. The excess of primary antibody was removed by three washing steps with washing buffer (PBS, 0.1% Tween 20, 0.1% BSA). Subsequently, the membranes were incubated with peroxidase-labeled secondary anti-goat antibodies at a dilution of 1:20,000 at room temperature for 1 h. Protein bands were visualized by chemiluminescence using the ECL detection system (GE Healthcare). As a loading control, the expression of β-actin was analyzed after stripping the blots with Re-blot Plus solution (Merck-Millipore, Darmstadt, Germany) according to the manufacturer’s manual. Pictures were captured by the INTASR chemiluminescence detection system (Intas Science Imaging Instruments, Göttingen, Germany). The intensity of bands was quantified through densitometry with the Gel-Pro Analyzer 4.0 software (Media Cybernetics, Silver Spring, MD, USA). 

### 4.11. Reactive Species Detection by DCF Fluorescence

To detect overall oxidative and nitrosative stress, cells were seeded onto 96-well coated black plates. Before addition of test compounds, cells were pre-incubated with 10 µM dichlorodihydrofluorescein diacetate DCFDA-H_2_ (Invitrogen, Karlsruhe, Germany) for 40 min at 37 °C. Plates were analyzed at 480/520 nm excitation/emission using the fluorescence reader Victor2 1420 Multilabel Counter (Perkin Elmer, Fremont, CA, USA). Each condition was analyzed at least in duplicate. Data were expressed as a % of untreated cells.

### 4.12. Lipid Droplet Detection

INS1E cells were exposed to 500 μM of PA or OA for 24 h. Cells were trypsinized and fixed in 1% paraformaldehyde for 15 min at room temperature. Thereafter, cells were stained with Oil Red O solution (Sigma-Aldrich, Munich, Germany) followed by DAPI staining, and washed twice with PBS (phosphate buffered saline). Lipid droplet formation was analyzed using a Cell^R^/Olympus IX81 inverted microscope system (40× objective, Olympus, Hamburg, Germany). The area within the cells was quantified by the use of Olympus xCellence Rt software (Olympus, Hamburg, Germany) at 546 nm excitation and 580 nm emission. For each conditions five to seven randomly selected images, (each containing 30 to 100 cells) were used to quantify the proportion of the lipid droplet area to the total cell area with the phase analysis module of the xCellence Rt software.

### 4.13. Data Analysis

All data are expressed as MEANS ± SEM. Statistical analyses were performed using the Prism analysis software (Graphpad, San Diego, CA) using *t*-test or ANOVA followed by Bonferroni, with *p* < 0.05 considered statistically significant.

## Figures and Tables

**Table 1:** Primers used in real-time RT-PCR analysis.

Gene	FW	REV
*Rat B-Act*	GAACACGGCATTGTAACCAACTGG	GGCCACACGCAGCTCATTGTA
*Rat Chop*	CCAGCAGAGGTCACAAGCAC	CGCACTGACCACTCTGTTTC
*Rat Ire1*	TTCTACATCTGGCAGCGGGAGG	TTCCACTTGGTGATGCGCCC
*Rat Mnf2*	TCCAAGGTCAGGGGAATCAGCG	TGGTGGTGTGGCCAATCCCA
*Rat Phb2*	AAGGAGTCATGGTGCCAAA	GTGTCCGGCATCCACG
*Rat Plin2*	TCGTCTCTCAGCTCTCCTGT	TAGGTGGAGCTCACCAAGGG
*Human SPL*	ACGGCCTGGTGGCATTA	CTGACAATTGGGGATTCCC
*Human B-Act*	ATGGATGATGATATCGCCGC	TTCTGACCCATGCCCACCA

## Data Availability

All relevant data sets for this publication are available from the corresponding author under reasonable request.
